# A Retrospective Case-Control Study Evaluating the Bowel Preparation Quality during Surveillance Colonoscopy after Colonic Resection

**DOI:** 10.1155/2014/681978

**Published:** 2014-03-06

**Authors:** Stefano Pontone, Giovanni Leonetti, Antonietta Lamazza, Fausto Fiocca, Angelo Filippini, Gianfranco Fanello, Fabrizio Cereatti, Enrico Fiori, Rita Angelini, Gregorio Patrizi, Manuela Brighi, Simone Vetere, Angelo Antoniozzi, Daniele Pironi, Simone Manfredelli, Paolo Pontone

**Affiliations:** ^1^Department of Surgical Sciences, “Sapienza” University of Rome, Viale Regina Elena 324, 00161 Rome, Italy; ^2^Department Pietro Valdoni, “Sapienza” University of Rome, Italy; ^3^Department of Surgical Sciences and Organ Transplantation—P. Stefanini, “Sapienza” University of Rome, Italy; ^4^Department of Surgery E Durante, “Sapienza” University of Rome, Italy

## Abstract

*Purpose.* Bowel preparation for surveillance endoscopy following surgery can be impaired by suboptimal bowel function. Our study compares two groups of patients in order to evaluate the influence of colorectal resection on bowel preparation. *Methods.* From April 2010 to December 2011, 351 patients were enrolled in our retrospective study and divided into two homogeneous arms: resection group (RG) and control group. Surgical methods were classified as left hemicolectomy, right hemicolectomy, anterior rectal resection, and double colonic resection. Bowel cleansing was evaluated by nine skilled endoscopists using the Aronchick scale. *Results*. Among the 161 patients of the RG, surgery was as follows: 60 left hemicolectomies (37%), 62 right hemicolectomies (38%), and 33 anterior rectal resections (20%). Unsatisfactory bowel preparation was significantly higher in resected population (44% versus 12%; P value = 0.000). No significant difference (38% versus 31%, P value = ns) was detected in the intermediate score, which represents a fair quality of bowel preparation. *Conclusions.* Our study highlights how patients with previous colonic resection are at high risk for a worse bowel preparation. Currently, the intestinal cleansing carried out by 4 L PEG based preparation does not seem to be sufficient to achieve the quality parameters required for the post-resection endoscopic monitoring.

## 1. Introduction

Although there are different tests for colorectal cancer (CRC) screening, colonoscopy is the most effective to rule out precancerous colonic lesions and prevent cancer, removing the adenomatous lesions during endoscopic examinations [1]. Adequate bowel preparation (BP) is essential to properly examine the colonic mucosa [2] and to schedule correct follow-up colonoscopy intervals [3]. There are independent clinical risk factors for inadequate colon cleanliness [4] and several regimen factors that determine BP quality [5]. Thus, no bowel preparation method meets the ideal criteria for bowel cleansing before colonoscopy [6] and many different protocols have proved to be equally adequate [7–10]. When a localized CRC is diagnosed, surgical resection remains the mainstay of treatment and evidence suggest that a regular surveillance including colonoscopy is effective to prevent future adenomas or recurrences and to improve survival [11].

In some cases, as it happens often after left-sided resections, colonoscopy is associated with a shorter insertion time because of anatomical changes (sigmoid-descending junction resection) [12]. Surprisingly, endoscopy after resective surgery may be not so easy and effective as expected because of suboptimal bowel function [13, 14], adhesions [15], and poor BP [14]. This study compares two groups of patients in order to demonstrate that colorectal resection is another factor associated with a poor BP.

## 2. Methods

From April 2010 to December 2011, 351 outpatients underwent endoscopic evaluation at our department. Those patients were then splitted into two homogeneous arms. In 161 patients the indication was surveillance colonoscopy after colonic resection and 190 patients with other indications were enrolled in our retrospective study. This study meets the Helsinki criteria, and all patients provided written informed consent. Data collected were age, sex, bowel cleanliness, endoscopist expertise, and a history/method of other abdominal surgeries. Patients who had a previous colonic resection were eligible for inclusion in the first arm named resection group (RG) (Table 1) and patients with other indications (such as screening, followup after polypectomy, ematochezia, anemia, abdominal pain, bowel habits change, etc.) were included in the control group (CG). In the RG we classified surgical methods into left hemicolectomy (LH), anterior rectal resection (ARR), right hemicolectomy (RH), and double colonic resection. All patients followed a fiber-free diet three days before the test and a liquid diet at dinner on the day before, fasting over midnight. A 4 L polyethylene glycol (PEG) based preparation was adopted, starting at 5pm on the day before colonoscopy. All patients followed a 4 L PEG full-dose bowel preparation schedule as described below: 2 L from 4:00 to 6:00 pm and 2 L from 7:00 to 9:00 pm (250 mL every 15 min), the evening before colonoscopy. Colonoscopy procedures were performed between 8 am and 2 pm. Patients in both groups consumed at least 75% of BP.

Procedures were performed by nine skilled endoscopists. All examinations were performed under conscious sedation, with a mean dose of midazolam equivalent to 0.07 mg/kg. In our unit, we have standardized the BP assessment, and 4/8 pictures were taken to show the cecal intubation and the bowel cleansing. Colonoscopy was considered as complete if cecum was intubated and the appendiceal orifice and ileocecal valve were identified.

After the procedure, bowel cleansing was evaluated using a modified validated BP scale the Aronchick (A) scale: excellent (small amount of clear liquid with clear mucosa seen; more than 95% mucosa seen), good (small amount of turbid fluid without feces not interfering with examination; more than 90% mucosa seen), fair (moderate amount of stool that can be cleared with suctioning permitting adequate evaluation of entire colonic mucosa; more than 90% mucosa seen), inadequate (inadequate but examination completed; enough feces or turbid fluid to prevent a reliable examination; less than 90% mucosa seen), poor (repreparation required; large amount of fecal residue precludes a complete examination) [16]. Depending on the chosen bowel preparation scale, we considered three levels of preparation: A1-2 (including grades “excellent” and “good”), A 3 (including “fair”), and A4-5 (including “inadequate” and “poor”). Our choice was influenced by the surgical resection that does not allow the use of segmental scales [17, 18]. The statistical analysis of the results was performed with SPSS version 20.0 (SPSS Inc, Chicago, IL). Differences among proportions were assessed by the *χ* ^2^ test. Student's *t* test was used to evaluate differences in means. Two-tailed *P* values <0.05 were considered significant.

### 2.1. Study Limitations

In the RG, it was not possible to distinguish between early (≤1 year after surgery) and late (>1 year) follow-up colonoscopy. Similarly it was not possible to determine the time elapsed after surgery.

## 3. Results

A total of 351 consecutive outpatients (203 male, 148 female, age range = 20–84) underwent elective colonoscopy. Groups were comparable for sex, age, obesity rating (mean BMI = 24.8 RG versus 23.7 CG; *P* = 0.17) [19], and number of patients at risk for developing medication-induced constipation (opioid or tricyclic antidepressant drugs) (Table 2). Among the 161 patients in the RG, surgery was as follows: LH (37%), RH (38%), and ARR (20%) (Table 3). In addition, six patients had undergone a double colonic (5 pts) and transverse resection (1 pt). Most treatments were performed in “open” surgery (94%). The indications for colonoscopy in the CG were as follows: followup for previous polypectomy or therapy monitoring (33%), hematochezia (19%), screening for CRC (15%), abdominal pain (11%), familiarity for CRC (10%), altered bowel habits (8%), and anemia (4%). Among the 190 patients, 23.7% had a previous abdominopelvic surgery. No significant statistical differences between both groups were observed about caecal intubation rate, median procedure time (23 min), and withdrawal time (>7 min). Five tests, in RG, were not completed because of a stenosis due to recurrent cancer. In one case this obstacle was overpassed using an ultraslim gastroscope (4.9 diameter) and in two cases using a standard gastroscope. In CG, four patients did not receive a complete examination because of poor BP (3 pts) and tolerability (1 pt). There were no complications related to endoscopic examinations or drug use.

Inadequate or poor bowel preparation (4 or 5 according to Aronchick scale) was significantly more frequent among patients with previous bowel resection regardless of the type of surgery performed (Table 2). The small number of ARR did not allow a reliable comparison on the quality of BP compared to other surgical treatments. On the contrary, no significant differences (38% versus 31%, *P* value = 0.59) were detected between RG and CG in the intermediate score (3 according to the Aronchick scale), which represents a fair quality of bowel preparation. Even more specifically, in scores 1 and 2 Aronchick, there were no significant differences between patients who underwent left or right hemicolectomy. Only 29 patients had a diagnosis of adenoma in the RG compared to 62 patients in the CG. Thus, the adenoma detection rate (ADR) was significantly different between the two groups as reported in Table 2.

## 4. Discussion

The colonoscopy-based surveillance after surgery for CRC represents the most advantageous choice in order to detect recurrence or metachronous neoplasia [20]. However, despite the diagnosis of recurrence is effective, the great advantage of surveillance seems to be related to the early diagnosis of metachronous adenomas in this high risk population. Moreover, surveillance colonoscopy in symptomatic patients within a year after surgery has limited value in terms of ADR [21] and a significant proportion of follow-up colonoscopies is performed too early according to current guidelines. Although in our study it was not possible to quantify the “weight” of early followup, this factor, along with poor BP, can probably interfere with the ADR detected in RG resulting in a lower detection rate.

Like in screening colonoscopies, a poor BP may impair also during a surveillance program the effectiveness of endoscopy. Today, the standard BP is represented by the 4 L PEG based preparation administered, when possible, with a split-dosage regimen [5, 22]. However, also this scheme of BP does not fully meet all required characteristics to achieve the optimal preparation.

This is particularly true, if we consider several independent predictors of inadequate bowel preparation, such as noncompliance with preparation instructions (stroke or dementia) or factors related to possible colonic dysmotility [23] and history of diabetes, hysterectomy, or colorectal resection [24]. Recently, the Lim study [14], which is the only study in the literature that addresses BP in postresection population, investigating the impact of previous gastric or colonic resection on bowel preparation for colonoscopy, asserts that resected patients (either stomach or colon) had worse BP quality. What Lim reported in his series of 92 patients was reinforced in our study, where a satisfactory BP was achieved only in 56% of cases. Considering the values disparity, we can postulate that the 27.2% of gastric resected patients included in the Lim series have a significant impact on the final result, even “underestimating” the reports outcomes.

Bowel dysfunction was evident after surgery [25], but the reason for a high percentage of poorly cleaned examinations in colonic-resected patients remains unclear. Some factors, such as adhesions syndrome, functions of colon mixing and cleaning alteration, and flexibility reduction, have been proposed. The fact is that the 4 L PEG preparation, in these cases, has important limitations. How these limitations are due exclusively to surgery and how, on the other hand, is the influence of the preparative regimen has not yet been investigated. In fact, there were no significative differences according to previous surgical procedures. In our study, on the contrary of what was logically expected on the influence of the ileocecocolonic segment [26], patient with left or right hemicolectomy had the same cleaning results. Thus, the minor role of ileocolonic junction in the transit of solid contents would be partly confirmed [27]. If the detection of recurrent cancer, metachronous cancers, and adenomas is the first goal of colonoscopy surveillance [28], then we can understand how important it is to achieve a proper bowel cleansing before endoscopy. However, this topic has been addressed in only one study [14], while investigations should be carried out to understand whether the BP should be the same for both resected or not resected patients. Poor functional results after resectional surgery are related to poor BP. Our study raises some questions about the impact of high volume PEG based BP in patients undergoing colonic resection.

## 5. Conclusion

The factors leading to poor functionality after colic surgery may also hinder the BP. Also additional factors not yet investigated, such as the length of the colon resected and the type of radiotherapy, may influence the effectiveness of the preparation. More studies are still needed to evaluate the efficacy of BP in resected patients. Currently, a satisfactory preparation regimen for these patients is missing.

## Figures and Tables

**Table 1:** Resected group inclusion and exclusion criteria.

Inclusion criteria	Exclusion criteria
Patients with previous colonic resection	Other types of abdominopelvic surgery

At least 75% of BP consumed	Serious medical conditions
	Cerebrovascular disease or dementia
	Inflammatory bowel disease

BP: bowel preparation.

**Table 2:** Patients characteristics and bowel preparation score.

	RG	CG	*P* value
Patients	161	190	
Age range	36–84	20–82	
Male	86	117	NS
Mean BMI	24.8	23.7	NS
Mic	15	21	NS
Caecal intubation rate (%)	97	98	
ADR (%)	18	32.6	0.002
Aronchick 1-2	9 + 20 (18%)	70 + 38 (57%)	0.000
Aronchick 3	61 (38%)	59 (31%)	NS
Aronchick 4-5	58 + 13 (44%)	19 + 4 (12%)	0.000

RG: resected group and CG: control group. ADR: adenoma detection rate; BMI: body mass index. Mic: number of patients at risk for developing medication-induced constipation.

**Table 3:** Bowel preparation evaluation in resected patients.

Surgery	pts	Aronchick 1–3 (%)	Aronchick 1-2 (%)
RH	62	51	16
LH	60	53	17
ARR	33	67	24
Other	6	—	—

LH: left hemicolectomy; RH: right hemicolectomy; ARR: anterior rectal resection.
